# Perceptions of Daily and On-Demand HIV Pre-Exposure Prophylaxis and Digital Adherence-Support Needs Among Cisgender Men in Brazil: Qualitative Interview and Focus Group Study

**DOI:** 10.2196/66848

**Published:** 2025-10-02

**Authors:** Lorruan Alves dos Santos, Alexandre Grangeiro, Paula Massa, Marcia Thereza Couto

**Affiliations:** 1 Departamento de Medicina Preventiva, Faculdade de Medicina FMUSP, Universidade de São Paulo, Av. Doutor Arnaldo, 455, Cerqueira César, São Paulo, 01246903, Brazil, 55 1130617086

**Keywords:** pre-exposure prophylaxis, HIV, sexual and gender minorities, digital health, qualitative research

## Abstract

**Background:**

Pre-exposure prophylaxis (PrEP) is a proven effective preventive method in reducing the risk of HIV infection. While daily PrEP is well-established, the on-demand regimen remains less accessible, despite offering advantages, such as a reduced pill burden and lower risk of side effects, particularly for those with variable sexual activity patterns.

**Objective:**

This study explored, from the perspective of PrEP use trajectories, how cisgender men in Brazil choose and adhere to daily or on-demand regimens, and identified key user-informed requirements for designing a digital tool to support on-demand PrEP use.

**Methods:**

A qualitative study was carried out between July 2022 and December 2023 in 5 Brazilian cities. Overall, 19 individual interviews and 5 in-person focus groups were conducted (N=47). The sample was diverse by age, education, race/color, time of PrEP use, and experiences of treatment failure. The median age of the interviewees was 34 (IQR 30‐36.5) years; 10/19 were <35 years and 9/19 were ≥35 years. The majority had completed higher education (15/19, 79%). The thematic saturation criterion was used, and the thematic analysis was conducted by 2 independent researchers with periodic consensus.

**Results:**

Participants perceived oral PrEP as highly effective in reducing HIV-related anxiety. On-demand PrEP was valued for its lower pill burden and a perceived lower risk of adverse effects. However, participants reported that adherence to this regimen demands considerable discipline and organization, posing a significant barrier. In this context, a support app was not met with mere acceptance but was conceptualized as a “cognitive offloading” tool, capable of transforming the burden of managing the 2+1+1 schedule into a positive and manageable task. Suggested functionalities included pill reminders, appointment scheduling, and geolocation of postexposure prophylaxis services. Nevertheless, significant concerns about data security and privacy were raised, with participants proposing that the state should manage the app to ensure confidentiality.

**Conclusions:**

Findings highlight clear generational differences in how cisgender men perceive daily and on-demand PrEP. Older participants draw on memories of the early AIDS crisis, whereas younger men situate PrEP within app-mediated sexual cultures. Importantly, they agree that the on-demand regimen requires not only greater discipline but also a learning process to incorporate and normalize its episodic dosing into everyday life. The positive reception toward a digital adherence support tool demonstrates potential public health value in such technologies, provided privacy and data security concerns are effectively addressed. Efforts to improve adherence and expand access to on-demand PrEP could significantly benefit from incorporating user-driven insights into digital tool development.

## Introduction

Oral HIV pre-exposure prophylaxis (PrEP) consists of using 2 antiretroviral drugs (emtricitabine and tenofovir) to reduce the risk of HIV infection in unprotected sexual exposures. Currently, there are different ways of taking PrEP, the most common being daily PrEP, which consists of daily ingestion of the medication for an indefinite period. Another approach is on-demand PrEP, an event-driven dosing regimen requiring 2 tablets between 2 and 24 hours before potential sexual exposure, followed by 1 tablet 24 hours and another 48 hours after the initial double ingestion (the 2+1+1 schedule) [1,2]. Several clinical and demonstrative studies have already demonstrated the safety, efficacy**,** and acceptability of PrEP among gay, bisexual**,** and other men who have sex with men in both forms of use [3-6]. However, on-demand PrEP is still significantly less available worldwide compared to daily PrEP, which undermines its accessibility [7].

Previous studies have already attested to the perception of daily PrEP users regarding the safety and efficacy of prophylaxis for HIV prevention, in addition to the feeling of greater tranquility in their sexual lives, increased self-esteem, and reduced anxiety related to the risk of HIV infection and shame, among others [8,9]. However, there is a lack of in-depth knowledge on the perceptions of on-demand PrEP users in Brazil, given its recent incorporation into the scope of the Brazilian Unified Health System (Sistema Único de Saúde, SUS in Portuguese) in 2023 [10].

Understanding how on-demand PrEP users perceive and symbolize prophylaxis use is crucial to developing effective health information-sharing strategies. These approaches need to reach the target populations of sexually transmitted infections (STIs) and HIV preventive methods, who are often in situations of greater social vulnerability. Since such susceptibility manifests in individual, social, relational, and programmatic dimensions, it is possible to promote greater adherence and effectiveness in sexual health strategies by adapting communication approaches to users’ perceptions and symbolisms.

Although the World Health Organization has recommended the use of event-driven PrEP since 2019, its implementation remains uneven across and within countries [11]. In Brazil, event-driven PrEP remains significantly underused. Barriers to its implementation include a lack of provider training and institutional support, as well as perceptions that this regimen requires greater user discipline [12,13]. Studies have shown that health care professionals often express greater confidence in prescribing daily PrEP, particularly in settings where service protocols are optimized for daily use [7,11,12]. Moreover, adoption patterns reflect broader structural inequities, with higher uptake of event-driven PrEP in high-income populations and countries, while individuals from lower-income backgrounds, who may benefit greatly from flexible regimens, face additional informational and access-related barriers [14]. Understanding these dynamics is crucial for informing effective and equitable implementation strategies.

On another front, with the advancement of digital technologies increasingly integrated into people’s daily lives, smartphone apps have the potential to stand out as essential tools to support PrEP users [15,16]. These apps may help ensure consistent adherence to prophylaxis, representing a crucial ally in prevention and sexual health promotion. However, initiatives exploring the possibilities of these new technologies and the acceptance of PrEP users are still in their infancy and almost nonexistent for on-demand PrEP [17]. Further, more research is needed to fully understand how these tools can be optimized and accepted by the community.

In light of that, this qualitative study aimed to understand, through the lens of users’ PrEP trajectories, the strategies adopted by cisgender men in Brazil to choose between daily and on-demand PrEP regimens, maintain adequate adherence, and manage episodic use. Furthermore, the study explored user-driven insights into the design and acceptability of a hypothetical smartphone app, with the explicit intention of identifying practical features and privacy considerations that could enhance the real-world applicability and effectiveness of digital adherence support tools for on-demand PrEP.

## Methods

### Overview

This is a descriptive qualitative study conducted within the fourth phase of the Combina! Study, a Brazilian multicenter study that demonstrated the effectiveness of HIV PrEP and combined prevention—defined as the integrated and simultaneous use of multiple HIV prevention strategies, including condoms, regular HIV testing, postexposure prophylaxis (PEP), PrEP, and other preventive practices—in the context of different sexual health services of the Brazilian Unified Health System from 2015 to 2023. It was performed in 5 Brazilian cities and with various methods of care, namely 2 HIV outpatient clinics (Ribeirão Preto and Porto Alegre), 2 testing and counseling centers (São Paulo and Curitiba), and an infectious diseases hospital (Fortaleza) [18]. The qualitative investigations of the Combina! Study fourth phase focused on the motivations for seeking and choosing event-driven PrEP, considering the reasoning for starting and stopping treatment, experiences with adverse effects, the strategies adopted to ensure adherence, the adequate use of medication, and the use of apps and other digital tools to support adherence to prophylaxis.

In this study, we analyzed data from 19 individual in-depth interviews with gay, bisexual**,** and other men who have sex with men from the 5 health services participating in the study and 5 focus group (FG) sessions with a total of 47 gay, bisexual**,** and other men who have sex with men in follow-up at the site in São Paulo/SP. Potential participants were initially identified by research professionals based on their history of taking daily and on-demand PrEP regimens. Eligible individuals were approached by researchers, informed about the study objectives, and invited to participate voluntarily. Recruitment included ensuring diversity in key sociodemographic characteristics (age, education, and race/ethnicity), PrEP use patterns (duration and adherence), and experiences of PrEP failure.

The semistructured interview and FG guides enabled further exploration of experiences of PrEP use in general, with a special focus on on-demand PrEP. Topics covered the sociodemographic profile, the PrEP usage trajectory (including information sources and previous experiences), and adherence strategies for the 2+1+1 regimen. Factors influencing the decision to initiate a regimen (such as partnership type, predictability of sexual relations, and substance use), methods used to remember taking the initial and final doses, and actions taken in response to missed or delayed doses were investigated. Perceptions regarding the use of digital technologies as an adherence support tool were explored, including desired functionalities and the feasibility of integration with existing apps. Finally, the study sought to understand the impact of PrEP on users’ daily lives and their perspectives on the characteristics of an ideal health service for PrEP provision. The interview and FG guides are available in the Multimedia Appendix 1.

All individual interviews were conducted via video calls, while FG sessions were held in person in safe and private locations. Interviewers and moderators took notes in field diaries to contextualize the information acquired during the interviews and FGs. The recordings were transcribed, reviewed, and deductively categorized in MAXQDA software version 2024 (VERBI Software GmbH). The final number of interviews and FG sessions was determined based on the criterion of theoretical saturation [19,20]**,** a principle widely used in qualitative research. This criterion guides the decision to cease data collection once sufficient thematic recurrence is observed to adequately address the research question. Saturation is conventionally considered achieved when participants’ narratives no longer yield new elements or pertinent information. In this study, the authors confirmed saturation following a review and preliminary analysis of the conducted interviews. We used fictitious names to ensure that participants remained anonymous.

The FGs were formed mainly taking into account the cohesion of each group about the criteria of time diversification of PrEP use, adherence history, age range, and education, being: (1) FG1: participants with a history of low adherence to PrEP on demand and/or failure in the use of PrEP; (2) FG2: participants less than 30 years old and with up to complete high school; (3) FG3: participants less than 30 years old and with complete higher education or more; (4) FG4: participants more than 35 years old and with complete higher education or more; and (5) FG5: participants more than 35 years old and with complete high school or less. Each FG was initially designed with a targeted participant profile, aiming to ensure internal cohesion based on age group, education level, duration of PrEP use, and adherence history. However, in practice, some participants who were originally invited to join a specific group were unable to attend at the scheduled time. These individuals proactively requested to be reassigned to other groups, believing that their experiences with PrEP use and participation in the study were valuable and needed to be shared. As a result, a few participants (n=4) were included in groups whose overall sociodemographic composition differed from their own profile. This reallocation, however, did not present any methodological issues. In qualitative research, such flexibility is both acceptable and expected, as it preserves the richness and relevance of participants’ narratives while respecting ethical and logistical aspects of data collection.

The analysis followed the main assumptions of the thematic analysis [21] based on the following steps:

1. Immersion in the contents of the interviews and FGs to capture an overall view and the specific aspects of the testimonies
2. Identification of the emerging empirical categories and categorization of the testimonies
3. Preparation of a preliminary synthesis with analytical sensitivity to the specific social markers of difference and the criteria used for sample diversification
4. Review of the scientific literature and discussion of the findings from the preliminary synthesis
5. Elaboration of the final interpretative synthesis.

Thematic analysis was supported by MAXQDA software (version 2024). To ensure analytical rigor, the process was conducted collaboratively by 2 researchers, including the corresponding author (LAS). Both researchers independently performed the initial coding of the transcripts. Subsequently, they held regular meetings to compare codes, discuss emerging themes, resolve discrepancies through consensus, and refine the final coding framework that guided the analysis.

### Ethical Considerations

This study was conducted in accordance with all requirements established by Resolution 466/2012 of the National Research Ethics Commission and Resolution 510/2016 of the National Health Council of Brazil [22,23]. The study protocol was previously approved by the Ethics Committee of the Faculty of Medicine at the University of São Paulo under review number 3.438.329/2019. All eligible individuals were informed of the research objectives, procedures, and ethical aspects, including assurances of confidentiality, and provided written informed consent prior to participation. To protect privacy and ensure confidentiality, all data were deidentified, and fictitious names were used in the presentation of the results. Compensation to cover participation-related expenses, valued at BRL 40 (US $7.78 on January 16, 2023), was offered to participants of the in-person FGs. Participants in the individual interviews, conducted remotely, did not receive financial compensation. This paper contains no images or any other information that would allow for the individual identification of participants.

## Results

### Overview

Most of the participants interviewed were receiving clinical follow-up care at the São Paulo service (n=10), and the others at Fortaleza (n=4), Porto Alegre and Ribeirão Preto (n=2 in each site), and Curitiba (n=1). In total, 6 of the 19 interviewees considered themselves lower middle class, 5 as middle class, 4 as upper middle class/upper class, and 4 did not respond. Regarding race/skin color, 13 participants self-identified as White, 5 self-identified as mixed-race, and 1 self-identified as Black. Regarding the age group, the median age of the interviewees was 34 (IQR 30‐36.5) years, with 10/19 participants being younger than 35 years and 9/19 participants aged 35 years or older. Regarding education, most participants (n=15) had at least completed higher education; 2 were enrolled in higher education, and the 2 had finished high school. The interviewees’ characteristics are similar to those of the current profile of PrEP users in Brazil regarding education, race/skin color, and income [24].

Table 1 displays the demographic information of the interviewed participants, and Table 2 summarizes the sociodemographic information of the interviewees.

**Table 1.** Sociodemographic profile of interview participants (n=19; 5 Brazilian cities; 2022-2023).

Codename	Age (years)	Race/color	Education	Professional activity
Allan	34	Mixed-race	Completed higher education	Financial and Administrative Sector
Carlos	28	White	Incomplete higher education	Arts and Communication
Enzo	51	Mixed-race	Completed higher education	Health
Ernesto	43	White	Completed higher education	Financial and Administrative Sector
Guto	37	White	Completed higher education	Health
Gilvan	29	White	Completed higher education	Arts and Communication
Jileandro	37	White	Completed higher education	Unemployed
Jamerson	31	White	Completed higher education	Financial and Administrative Sector
João	53	Mixed-race	Completed higher education	No information
Jorge	23	Mixed-race	Incomplete higher education	Financial and Administrative Sector
James	58	Black	Completed higher education	Education and Research
Lucas	31	Mixed-race	Completed higher education	Technology and Sales
Liandro	32	White	Completed higher education	Health
Lauro	34	White	Completed higher education	Education and Research
Lisandro	37	White	Completed higher education	Arts and Communication
Marcelo	38	White	Completed higher education	Arts and Communication
Mauro	34	White	Completed higher education	Financial and Administrative Sector
Viana	25	White	Completed higher education	Health
Wellington	35	White	Completed higher education	Health

**Table 2.** Aggregated sociodemographic characteristics of interview participants by frequency and percentage (n=19; 2022-2023).

Characteristic and category		Value, n (%)	
Age group (years)			
<35		10 (52.6)	
≥35		9 (47.4)	
Race/color			
White participants		13 (68.4)	
Mixed-race participants		5 (26.3)	
Black participant		1 (5.3)	
Education			
Completed higher education		15 (78.9)	
Incomplete higher education		2 (10.5)	
Completed secondary education		2 (10.5)	
Professional activity			
Health		4 (21.1)	
Arts and Communication		4 (21.1)	
Financial or administrative sector		4 (21.1)	
Education and Research		2 (10.5)	
Technology and Sales		1 (5.3)	
Unemployed		1 (5.3)	
Information not provided		3 (15.8)	

To contextualize the participants’ perceptions, their PrEP use trajectories were analyzed. The majority chose to use PrEP, adopting only 1 modality. The preferred regimen was event-driven, as a first choice (10/19) or switched to it from a daily regimen (5/19). A minority (3/19) did the reverse, adopting daily use after finding the 2+1+1 schedule impractical for a more frequent and unpredictable sex life. The preference for the on-demand regimen was dominant (12/19) and motivated by a lower pill burden and its suitability for a sporadic or planned sexual routine. However, for those who preferred the daily regimen (4/19), the main reason was linked to practicality and safety in contexts of greater unpredictability. On the other hand, a fluid approach, which means users making “microswitches” between the 2 modalities, was also an option for a small group. For them, the preference was more related to specific moments of life, like travelling, than the regimen or the medication effects.

Cell phone alarms were the main adherence strategy used by participants. Common challenges among event-driven users included forgetting the final doses and the logistics of the initial dose for unplanned encounters, which led 1 participant to need PEP. The detailed information regarding these individual trajectories and experiences is displayed in Table S1 in Multimedia Appendix 2.

### Perceptions About PrEP and the Event-Driven Regimen

Most participants considered PrEP to be an effective HIV prevention measure, which reduces anxiety and fear related to the risk of infection by the virus. Memories of the AIDS crisis in the first decades of the epidemic served as a powerful reminder of the importance of prevention, especially for older participants who began their sexual lives in the early years of the epidemic, as illustrated in the following statements.

*[...] My first sexual experience was occurred back in the 90s; I was terrified of contracting HIV and getting sick. So my entire sex life at the beginning always considered condoms [...]. Then, when PrEP came along, and changed that scenario. I could try something else now, and I think that today, this generation that has overcome HIV. Right? PrEP gives you that feeling. Right? However, it is just a feeling, too. At the same time, there are other STIs. I always used the combination, the condom with PrEP, but then there comes a time when you think: “Wow, there are a series of possibilities that I can try, and PrEP gives you that real possibility because you know you will not get HIV and the other STIs are treatable.” So, that feeling was very liberating when it came to having sex*.

[FG1]

*[...] I am 56 years old, coming from a generation that reached the sexually active age in the middle of the AIDS epidemic so to this day, I still talk to my doctor about it. Yet, she often states,* “*Look, relax a little more.” I got used to it, you know. I was always afraid since I lived in a time with a lot of stigma, etc, but lately, I think I am managing to use PrEP well, you know, and relax a little more in relationships**.***

[FG3]

New challenges in the context of HIV prevention have emerged with the advent and consolidation of so-called dating apps, such as Grindr (Grindr LLC), for younger people, although not exclusively. The fear of possible infection by the virus in a scenario where interactions are frequent and diverse motivated most participants to seek PrEP. Among participants, the information shared through dating apps was the primary source of information. This is illustrated in the following example:

*Then I started using apps. Until then I did not have dating apps like Grindr, so I always saw PrEP, PrEP [...] I thought: what is PrEP? Then I started talking to friends who told me: “Oh, PrEP is a system like that.” Then I went to [...] and looked it up [name of service] and then health carers said they had an on-demand program, which I thought would be interesting to join. [...] That is how I joined. I was on kind of dangerous ground, risking things [...] I thought it was not cool. Then, I started using PrEP and ended up having less sex, I do not know why [...]*

[FG3]

Our results reveal that participants’ general perceptions of PrEP position it as an element for overcoming HIV. Whether through echoes of challenges faced in the past or as a response to current risks, PrEP represents a bridge from the memory of periods of greater fear, uncertainty, and lack of effective prevention/care technologies to modernity. In this context, prophylaxis represents the reduction of anxiety and concern related to sexual health in a situation where new scripts of affective-sexual interaction emerge, resulting in a feeling of greater sexual freedom.

*Even though we know that PrEP does not prevent other things, I believe that for many people, it is not just about the security of phew; I am aiming at more security. Still, you also find a way to have greater sexual freedom to discover yourself in other ways. I believe that for me, at least, it had that effect.*

[FG5]

In parallel, some participants reported that third parties (ie, friends and sexual partners) consider them responsible and diligent in preventing HIV. However, some view PrEP use negatively, stigmatizing these individuals as promiscuous or even as potential disseminators of other STIs, such as syphilis, which PrEP does not prevent.

*But I think you’re right; I think most people believe that those who use PrEP are promiscuous [...]*.

[FG1].

This contrast in opinions highlights the complexity of the stigma surrounding PrEP and the importance of better understanding the diverse sociosexual positions of its users.

Event-driven PrEP, specifically, was perceived by participants as having some advantages over the daily regimen, such as reducing the number of pills consumed, which, in turn, from the participants’ perspective, reduces the risk of short- and long-term medication-associated adverse effects associated with continuous treatment:

*I think you put less strain on your body [in the on-demand PrEP regimen]*.

[FG4]

This approach would be particularly advantageous for individuals who reported not engaging in intense sexual activity, allowing them to use their medication more targeted and consciously.

Furthermore, event-driven PrEP stood out as ideal for people with professional and personal lives marked by alternating periods of intense work activity and others with more leisure and, consequently, sexual activity. That, in turn, allows the medication to be necessary during times of more frequent sexual relationships.

However, many participants reported the unpredictability of discipline and organization in the on-demand regimen compared to daily use. In most of the analyzed situations, this is because on-demand PrEP requires attention to the times of medication use, that is, waiting for the necessary time to be able to initiate risky sexual practices and the need to take medication for a more extended period even after the sexual scene has ended (+1+1). An example is illustrated below:

*So ... are you organized, or are you rule-based? I think that you need to be very disciplined first of all, even more than you need to reflect on your sexual routine.*

[FG5] 

### Exploring the Idea of a Hypothetical App to Support Event-Driven PrEP Adherence

All interviewees and FG participants were asked to reflect on the relevance and acceptability of a hypothetical smartphone app that primarily supports on-demand PrEP adherence and use. During the individual interviews and FGs, a brainstorming process was encouraged about possible functions and the ideal scope of this fictional app, as the entire group is already aware of this type of digital technology. The collaborative nature of the FGs allowed fruitful discussions, leading to in-depth considerations about the pros and cons of each proposal. Ideas could also be presented and discussed in the private environment of individual interviews without the risk of being judged or confronted with counterarguments by other participants.

The analyses indicated a consensus on the potential uses and benefits of the hypothetical app as an additional resource to support adherence and management of on-demand PrEP use. Some participants, however, did not consider themselves as the primary beneficiaries or in need of these resources but recognized that they could help other prophylaxis users who need more support to manage their use. For example, people who need friendly reminders of when to take pills and when to stop the regimen. In addition, participants emphasized that a key differentiator for the app would be its ability to adapt to different sociosexual contexts and patterns of PrEP use. For instance, some users reported irregular or episodic sexual activity, requiring more flexible tools to manage on-demand PrEP, while others needed features aligned with daily routines. Participants also mentioned the importance of personalizing notifications and language used in the app to reflect different levels of disclosure and comfort regarding sexuality, sexual orientation, and HIV prevention. This flexibility was seen as a way to go beyond generic pill reminder functions and offer tailored support that respects users’ lived realities.

In summary, perceptions about the acceptability of a digital tool to support adherence to on-demand PrEP vary between 2 types of users. The first group does not see the need for additional support beyond the tools they already use, such as smartphone alarm apps, and does not consider themselves the primary beneficiaries of new tools. The second, more enthusiastic group values an app with multiple features and functionalities that integrate with different services and is less concerned about revealing their PrEP user status. This group also appeared more creative in FG sessions, suggesting multiple features and integrations, even though some do not personally want new tools.

Carlos, for example, a 28-year-old PrEP user who lives with his family and whose sexual orientation has been known to them for more than 15 years, expressed his desire for an app that went beyond specific support for on-demand PrEP management. In his words, he “dreams” of an app that integrates more aspects of his sexual health and offers a more “holistic” approach to sexual health and behavior.

*[...] It would be a dream to have an app that could coordinate, and bring everything together. For example, if I could mark that I started using PrEP on a particular day because I am going to have an exposure or event. It would be great if I could receive alerts, such as: “It is time to have your health checked. Your last exams were on such a day. It would be good for you to do them again.” That would be interesting [...] something more holistic about the individual behavior [...]*

[Carlos]

On the other hand, Lisandro, who has “no shame,” is not concerned about the hypothetical apps’ level of security and privacy. However, he recognizes that other users in different situations could need more privacy and security to feel comfortable using the app.

*Interviewer: And regarding security, what does this application need to guarantee security and privacy?*

*Lisandro: It depends a lot on the person. I have no qualms about hiding anything, but some people may feel uncomfortable and don’t want to expose themselves. However, for me, it would be indifferent.*

On the other hand, some users who are more wary of revealing their sexual lives expressed concerns about integrations with other apps, with the health service, and with messaging apps, such as the creation of a chatbot on WhatsApp (Meta).

We organized the suggestions from the analyzed statements into 3 main categories for developing the hypothetical app. These categories are security and confidentiality measures, necessary functionalities, and presentation and layout. Security and confidentiality measures involve practices and technologies to protect users’ privacy and ensure their personal and health information security. Functionalities include tools, such as pill-taking reminders, integration with health services, and personalized support. Layout refers to the intuitive and attractive design of the app, ensuring a pleasant and efficient experience for the user. These categories, however, are interconnected and do not exist, from the participants’ perspective, in isolation or hierarchically positioned. Below, we describe these categories for explanatory purposes.

### Security and Confidentiality

Regarding security and confidentiality measures, many participants suggested that the State, through the Unified Health System (SUS), should assume responsibility for monitoring and managing services: “*It could be linked to the SUS, Conecte SUS, something like that*” (FG 3), which would guarantee even more reliability and transparency.

However, some participants expressed concerns about integrating data related to PrEP use with other health information from different databases that need to be audited and managed by public agencies, such as the SUS. They imagined a hypothetical scenario where the databases of health services, such as basic health units and specialized care services for STIs/HIV/AIDS, would be unified within the scope of the SUS, and sensitive data about sexual health would be available to all health care professionals indiscriminately.

*So, it may come up against the issue that I go to the cardiologist to take care of my heart, but I do not want to say that I am gay and take PrEP. And then in one of these things [the information] will cross [...] she already has the data collected by the SUS. If she is afraid, she will not even ask for PrEP; if she is scared of being discovered, she will not even go in...*

[FG 3]

There were concerns about integration into other apps, such as WhatsApp or Grindr. There is a fear shared by many that large corporations could collect and share sensitive user data.

*If I were a stalker or a hacker, and I wanted to know about someone’s sex life, I would get their WhatsApp number. Knowing that this tool exists, I could get their WhatsApp number, check if they have a PIX key [Brazilian instant payment system] registered to that number, and get their data and cross-reference it with the information from the chat app in a conversation. Suppose my boyfriend and I get his cell phone and see this conversation. I can identify it there, you know? I don’t know to what extent this is a problem; it is harmful, right? However, a person looking for discretion will not release this type of information on the internet. They will not integrate this information with large corporations, you know? And on Facebook and WhatsApp, only God knows what Meta is collecting there; if they see it, they do not see it.*

[Gilvan]

*[...] I would at least like to have a login and password so I can access my data there, whether one like it or not. In this context of having exams and everything else, and so on, I would be more cautious since my whole life is there. It is confidential data about your life. Anything that you treat confidentially needs additional protection, such as a username and password or something like that. It could be different: I opened it, it’s available here, and maybe even the logo is more discreet, not so much [...]*

[Mauro]

*[...] I think that if there were an app like that, the simpler it is, the more [...] individualized and less connected, that would work even without internet access, right, that has the manual there that you don’t need to be connected to 3G to load the information, better. If it even had the possibility of you entering a password or biometrics, no, but at least a password for you to type in to open it, it could also be cool*.

[Gilvan]

Some participants emphasized the importance of a discreet app, without colors or images indicating that it was an app about PrEP or sexual health, and, in addition, that it did not request excessive personal information and had security measures to limit access to the app for fear that third parties, especially family members:

*Ah, it’s kind of confidential, right? For example, I talk openly with my friends. Not with my family, because I say,* “*oh, bitch, right?” We’re afraid of being judged.*

[FG 2]

### Functionalities

The main features described by participants include (1) access to the calendar of upcoming scheduled medical appointments, with the ability to view and manage appointments directly through the platform, integrating with the health service’s schedule and enabling greater autonomy to reschedule appointments if necessary; (2) access to the medication dispensing history, allowing them to know how many pills they have in their possession and how many days are left until the stock runs out; (3) access to the results of the latest laboratory tests directly on the platform; (4) reminder functionality to ensure notification to retake tests at predetermined periods; (5) geolocation of the nearest PEP service, facilitating quick access to the necessary treatment in cases of event-driven PrEP failure.

*[...] So I would think that it has to be an application that works both online and offline; I would like there to be a space connected to where I take exams, and if this is not automatic, I would have to set it up to know when I return and have my information. This online business is also fundamental; people can choose whether to access WhatsApp or only use it on their cell phones*.

[FG3]

### Presentation and Layout

Regarding presentation and layout, participants suggested a design of the hypothetical app to satisfy different levels of technological ability, ensuring accessibility and availability for everyone, regardless of their familiarity with smartphones, mobile apps, and the internet. Participants pointed out the essentiality of personalizing its use according to specific needs. One example of customization suggested by participants is the individualized configuration for each event-driven PrEP user, according to their sexual routine. The suggestion is that by including information, such as expected days and times for sexual intercourse in the coming days, the app would recommend the best regimen (daily or on-demand) for each day and would advise when to take the last pill, facilitating the transition between the daily and on-demand regimen. In this way, in the end, it would be possible to know which days it would be safe to go without taking a pill.

From the following report, we can assume that the possibility of customization in the app’s presentation would also be desirable:

*It may be a bit prejudiced, but I don’t know the percentage of gays in Brazil who take PrEP or would like to take PrEP but are not out to their family or are not out at work or in any environment, college, or school. If a notification goes up to remind me from the PrEP app, and there’s a rainbow logo, and I’m in an environment where other people are watching, they’ll ask me why that rainbow is going up.*

[FG3]

The colors associated with the LGBTQIA+ (lesbian, gay, bisexual, transgender, queer, asexual, intersex, genderqueer and other sexual and gender identities) community or the icon of a PrEP pill, for example, could be optional or customizable by the user. In this way, those who fear discrimination when revealing their use of PrEP would not feel insecure when using the app, avoiding the risk of exposing their sexual orientation to people outside their intimate circle. This customization would allow users to adjust the app interface according to their preferences and needs, promoting a safer and more inclusive environment. In addition, the possibility of visual customization would contribute to a more comfortable and confidential user experience, encouraging adherence to treatment without fear of stigmatization.

## Discussion

### Principal Findings

This study explored the perceptions of cisgender men in Brazil regarding daily and on-demand PrEP, highlighting their motivations for regimen choice and their attitudes toward a potential digital adherence-support tool. Overall, participants viewed both regimens positively, emphasizing the reduction of anxiety related to HIV risk. On-demand PrEP, in particular, was valued for its adaptability to variable sexual routines and perceived reduction in medication-associated adverse effects. There was broad acceptance of a smartphone app as an adherence aid, contingent on stringent privacy and data security measures.

Overall, our results indicate that the participants have a positive perception of PrEP, broadly believing in the safety and efficacy of the method. Furthermore, many participants attributed a positive symbolic value to prophylaxis, highlighting its ability to reduce the negative emotional burden associated with sexual stigma and AIDS, especially in the first decades of the epidemic, a period that some call the “epidemic of meanings associated with AIDS” and that has effects to this day [25]. This finding relies on the high frequency of this perception among older participants and those who began their sexual lives in the context of a less intense HIV/AIDS epidemic.

On the other hand, young participants described PrEP as a valuable tool for navigating new modes of sexual and social interaction, especially those facilitated by dating apps and digital platforms. These interactions are often shaped by fast-paced, app-based dynamics, where decisions about partners and sex occur quickly and with limited prior connection. Some researchers have argued that these patterns reflect broader social trends influenced by individualism, market-oriented thinking, and structural inequalities, dynamics often referred to as neoliberal and colonial rationalities. Participants’ views reflected both empowerment through increased control over HIV prevention and concern about stigma and exposure in these digital environments [26-28].

Several studies have shown that messaging apps, such as WhatsApp, and social media platforms, such as Instagram (Meta) and TikTok (ByteDance), have played an increasingly important role in disseminating information about sexual health and new HIV prevention methods [29-32], including PrEP in the Brazilian context [33]. These digital channels offer a privileged reach to the most vulnerable populations that are difficult to reach [15,16,34]. In the literature, we find reports of policymaking studies that use social media and even artificial intelligence as tools to raise awareness for sexual and gender minorities through the creation of chatbots, scientific dissemination profiles, and dissemination of health information through prevention ambassadors involved in the creation of content about health and, more specifically, sexual health [30,32,34].

The stigma associated with PrEP perceived by participants has received increasing attention in the scientific literature as more people gain access to this form of prevention in Brazil and worldwide [35,36]. In a previous study [33], we investigated the dynamics between digital and in-person networks of gay, bisexual**,** and other men who have sex with men regarding the decision to use PrEP and the disclosure and publicity of this choice within and outside their social networks. We identified several motivations for users to disclose the use of prophylaxis to others, including (1) interest in prevention within the gay community, (2) the desire to distance themselves from stigmas related to AIDS and homosexuality, and (3) the possibility of increasing their sexual capital and “fuckability*.*” We also observed a widespread perception of the existence of a dichotomy between the “good” and the “bad” PrEP user, that is, between those socially seen as health-conscious and those who secretly aim to increase their sexual experiences regardless of the risk of other STIs.

Our results, in agreement with the scientific literature, reveal that the symbolic and cultural meanings of PrEP are anchored in the intersection of the ghosts of AIDS and sexuality stigmas in force at the beginning of the epidemic with contemporary sociodigital and sexual issues. Regarding the perceptions about the use of PrEP, for example, they can commonly be positioned somewhere on the spectrum between antagonistic symbolic and conceptual poles, ranging from the idea of a person who recognizes their risk of HIV/AIDS infection and who takes care of and protects themselves with the use of PrEP to the concept of promiscuity of a person who used to be protected by other methods and now wants to use PrEP to have unprotected sex [33,35,36]. Hence, we infer, based on empirical data, that in some cases, the sexual activities of some users may even be contrary to social expectations of so-called “good sex” [37], which does not mean that they are not planned and protected.

We found that on-demand PrEP was a better fit for some people’s sexual and HIV prevention routines than daily PrEP, but health concerns and the amount of pills used also influenced this. Our findings indicate that on-demand PrEP is perceived as a regimen requiring considerable discipline and organization, not only to initiate a dosing cycle but also to align the treatment with daily life and work routines. This highlights a significant barrier to the implementation and scale-up of on-demand PrEP, as the high cognitive and behavioral load placed upon users may compromise correct use and sustained adherence to the prophylaxis. This perceived difficulty explains the high acceptability of a digital support tool. An effective app would function not just as a reminder system but as a cognitive offloading tool, helping users manage the complex 2+1+1 schedule and mitigate anxiety related to potential adherence errors. We conclude that overcoming this “discipline barrier” is paramount to the successful expansion of on-demand PrEP, positioning digital tools as essential components, rather than mere accessories, in health programs.

Some of the on-demand PrEP users in our study revealed that the on-demand regimen offers valuable advantages over the daily regimen and that, by allowing them to reconcile work and research demands with moments of more significant sexual activity, it made them realize that the on-demand regimen is the one that best fits their sexual routines and the repertoire of combined HIV prevention methods that they have. In addition, on-demand PrEP, in the view of some participants, would offer lower risks of adverse effects associated with the medication and less impact on users’ physiology. Similar results were found in previous studies [38,39].

The discussion about the hypothetical app to support adherence to on-demand PrEP revealed broad acceptability of the tool among on-demand PrEP users, and even though some do not see themselves as the primary beneficiaries of the app, they wish it were developed, made available to their community, and managed by the state. This finding corroborates the hypothesis of community protection through the dissemination of knowledge about HIV prevention by users who decide to publicize the use of prophylaxis to third parties on dating apps [33]. These results also aligned with studies on creating and implementing digital strategies to support the continuum of care in PrEP [40].

The on-demand PrEP users interviewed are enthusiastic about thinking about how to balance the challenges of using this regimen flexibly through a customizable app. This hypothetical app could provide personalized reminders, medication information, access to sexual health resources, and emotional support. The challenge of remembering to take the pills daily is less remarkable, and overcoming it is feasible with common apps with the functionality to send notifications and reminders. Investing in downloading an extra app on their electronic devices, even if it means taking up gigabytes (GBs) of hard-earned space, seems to be, for on-demand PrEP users, a way to tip the balance of pros and cons in favor of the advantages when considering switching between oral PrEP use modalities.

The time investment in customizing an app is not considered onerous for most of the group. On the contrary, it opens up the possibility that it could be helpful to expand its functionalities by adding and unifying complementary forms of sexual health care in the app, such as frequent testing for other STIs and PEP. This expansion may indicate that despite having PrEP pills, the on-demand PrEP user is concerned about maintaining the testing frequency, which could be more flexible since users do not need, hypothetically, a new appointment in 120 days to renew their PrEP supply. In addition, it highlights the concern of needing PEP when the regimen is incorrectly applied, either because it was not possible to start the attack dose before sexual intercourse or due to failure in the +1+1 doses. Thus, there is a perception of a more significant advantage in not needing to use PrEP daily once this decision support for managing correct use accompanies the users and, consequently, leads to an increase in the perception of safety and protection.

Our results suggest that there are 2 groups of on-demand PrEP users based on their perception of their own sociosexual identity. One group includes people who are concerned about the publicity of their sociosexual orientation, and a second one who is excited and creative about the functionalities and scope of the hypothetical app. The management of sexual and AIDS stigmas, as well as their own safety needs, determines which group a given individual will position themselves in.

Further studies may address the question of how the anticipation of sexual and AIDS stigmas could destimulate the engagement with supporting apps as a strategy to minimize the risks of unwanted publicity about their PrEP use. That assumption indicates that the app could be less valuable to older and/or more vulnerable people.

The proposal to transform the app into a tool managed by the state through the SUS emerged as a partial solution to the security issue. Although it offers protection to users, reducing the perception of risk in the sale and circulation of private data, it also raises questions about the increased exposure of personal data about sexual health. For instance, health care professionals from other areas could access sensitive information, potentially generating prejudiced attitudes and experiences of embarrassment during care.

We believe that including this app in the offerings of “*Meu SUS Digital*” is a powerful and promising strategy because it strengthens access to and reliability of digital health services and guarantees the populational well-being to the detriment of commercial interests. However, following up the implementation is pivotal by training measures for health care professionals in general on stigma and prejudice in the field of sexual health.

This study has some limitations. First, participants were recruited primarily through convenience sampling in clinical settings, which may lead to bias toward individuals more engaged in health services. In addition, we did not systematically collect quantitative data on participants’ PrEP histories, such as the exact frequency of switching between regimens or the initial regimen chosen, which could have provided further context to their perceptions. Furthermore, despite efforts to ensure greater diversity, the sample predominantly included participants with a high level of education and urban residence—a profile similar to PrEP users in Brazil—which limits generalizability to populations that do not live in large urban centers and are less educated. Furthermore, because all data were self-reported, social desirability bias may have influenced participant responses, particularly regarding sensitive topics, such as sexual behavior and adherence. This potential phenomenon was likely less prevalent in individual interviews compared to FG sessions. However, the researchers responsible for conducting the FGs had extensive experience in leading discussions on sensitive topics and, when necessary, intervened to ensure respect among participants and a safe space for the expression and discussion of ideas.

Our findings reinforce PrEP's recognition as an effective HIV prevention tool that significantly reduces infection-related anxiety, corroborating previous literature. On-demand PrEP emerged as particularly valuable for users with variable sexual activity patterns, offering perceived advantages including reduced pill burden and lower risk of long-term adverse effects. However, further research is needed to understand how these advantages are perceived by daily PrEP users and those who chose alternative prevention strategies within the Brazilian context.

The broad acceptability of a digital adherence support tool reveals important implementation considerations. Participants conceptualized such tools as valuable "cognitive offloading" mechanisms for managing the complex 2+1+1 schedule, though effectiveness would depend on integrating three critical dimensions: practical dose management, stigma mitigation, and data security guarantees. Notably, state management through SUS was identified as essential for ensuring user trust and data protection. These findings suggest that successful scale-up of on-demand PrEP requires comprehensive digital support systems that move beyond generic reminders to address users' diverse sociosexual contexts and privacy concerns while coupling technological solutions with broader stigma-reduction efforts.

### Conclusion

This study revealed that cisgender men in Brazil perceive both daily and on-demand PrEP as effective HIV prevention tools, with on-demand PrEP particularly valued for its reduced pill burden and flexibility for variable sexual activity patterns. Participants demonstrated broad acceptance of a digital adherence support tool, conceptualized as a “cognitive offloading” mechanism to manage the complex 2+1+1 dosing schedule, contingent upon robust privacy protections and state oversight. Our findings indicate that effective digital adherence tools must integrate 3 critical dimensions, such as practical dose-management support, active stigma mitigation, and guaranteed data security. These user-driven insights suggest that policies aimed at scaling on-demand PrEP should prioritize co-design with end-users, embed privacy-first architectures, and couple technological solutions with broader stigma-reduction efforts to meaningfully enhance PrEP uptake and sustained adherence. Future implementation strategies must address the identified “discipline barrier” through comprehensive digital support systems that respect users' diverse sociosexual contexts and privacy concerns.

## Supplementary material

10.2196/66848 Multimedia Appendix 1 Interview and focus group scripts.

10.2196/66848 Multimedia Appendix 2 PrEP use trajectories, regimen preferences, and adherence experiences among 19 interview participants across 5 Brazilian cities (2022–2023). PrEP: pre-exposure prophylaxis for HIV.

## Abbreviations

FG: focus group
LGBTQIA+: lesbian, gay, bisexual, transgender, queer, asexual, intersex, genderqueer, and other sexual and gender identities.
PEP: postexposure prophylaxis
PrEP: pre-exposure prophylaxis for HIV
STI: sexually transmitted infection
SUS: Sistema Único de Saúde
