# Supplementary material for: Perceptions of Daily and On-Demand HIV Pre-Exposure Prophylaxis and Digital Adherence-Support Needs Among Cisgender Men in Brazil: Qualitative Interview and Focus Group Study
Source: J Med Internet Res. 2025 Oct 2;27:e66848. doi: 10.2196/66848 (PMC12492313; doi:10.2196/66848)
Supplement: Multimedia Appendix 1 [file jmir-v27-e66848-s001.pdf]

SEMI-STRUCTURED INTERVIEW SCRIPT

Socioeconomic Identification Block

- Name
- Date of Birth
- Self-identification
- Race/Skin color
- Gender Identity
- Sexual Identity/Orientation
- Self-perceived class*
- Housing Context
    - Who do you live with and are there conflicts?
    - Neighborhood context and access to resources like cable internet and cell phone signal
- Education Level

On-demand PrEP

- How long have you been using PrEP and what has your experience been like?
    - *Explore:*
        - Use of other prevention methods
        - Occurrence of adverse effects
- Strategies and resources for remembering to use on-demand PrEP
- What do you usually take into consideration when starting a new on-demand PrEP regimen?
    - *Explore:*
        - Type of partnership
        - Location of sexual relations
        - Predictability of sexual relations
        - Possibility of delaying the start of intercourse
        - Use of alcohol and drugs
        - Routine at the time of intercourse
- In which situations do you not use PrEP?
    - *Explore:*
        - Type of partnership
        - Location of sexual relations
        - Predictability of sexual relations
        - Possibility of delaying the start of intercourse
        - Use of alcohol and drugs and chemsex
        - Routine at the time of intercourse
- Tell me about an experience where you chose not to use PrEP.
- What do you do to remember to start an on-demand PrEP regimen?
    - *Explore:*

        - Electronic resources (apps, alarms, online calendar, etc.)
        - Analog/behavioral resources (pills in sight, associating with meals, etc.)
    - *Explore:* whether these resources are used only for PrEP or for other continuously used medications
    - *Explore:* the positive and negative aspects of each mentioned strategy
- How do you use on-demand PrEP when you have sex on multiple different days of the week?
- What do you do to remember to take the final pills of an on-demand PrEP regimen (+1+1)?
    - *Explore:*
        - Electronic resources (apps, alarms, online calendar, etc.)
        - Analog/behavioral resources (pills in sight, associating with meals, etc.)
    - *Explore:* whether these resources are used only for PrEP or for other continuously used medications
    - *Explore:* the positive and negative aspects of each mentioned strategy
- What do you do when you forget or delay a dose of on-demand PrEP?
    - *Explore:* behavioral changes and pill usage

New technologies to support on-demand PrEP use

- If there were an application to help remember to take on-demand PrEP, would it be useful for you?
    - *Explore:* if and how these resources would fit into the moments of starting, continuing, and finishing an on-demand PrEP regimen
- Imagine you were invited to give suggestions and ideas to a team developing an application to help PrEP users take their pills.
    - What ideas or suggestions would you give?
        - *Explore:*
            - Security
            - Privacy (camouflage, access password, etc.)
            - Internet consumption
            - Availability on different OS (Android, iOS, Windows, etc.)
            - Design (colors, style, etc.)
            - Online and offline?
            - User identification (does it need login?)
            - Standalone or embedded in another app (chatbot, for example)
- One possibility would be to implement these PrEP use support tools within existing applications.
    - What do you think of this idea?
        - *Explore:*
            - Which apps would be ideal (WhatsApp, dating apps, etc.)
            - How would the reminders work, a new tab...

        - What are the advantages and disadvantages of developing these resources as part of other apps?
- What aspects of PrEP use interfere with your daily life?
    - *Inhibit reports related to pill use and PrEP management and explore impacts on daily life.*

FOCUS GROUP SCRIPT WITH USERS FROM SÃO PAULO

Introduction (5 min.)

- General presentation of the research, reinforce research ethics information (confidentiality, academic purpose, etc.), reinforce that there are no right or wrong answers, we would like to hear all opinions (reinforce throughout the group).

Part One: Use of On-demand PrEP (20 – 25 min.)

- How did you learn about on-demand PrEP?
  - *Explore:*
    - Circulation of information on social networks from virtual and in-person peers
    - Quality of the information received
    - Valuations of on-demand PrEP (and differences compared to daily PrEP)

Part Two: Strategy for using On-demand PrEP (20 – 25 min.)

- What do you take into consideration when starting an on-demand PrEP regimen?
  - *Explore:*
    - Type of partnership
    - Location of sexual relations
    - Predictability of sexual relations
    - Possibility of delaying the start of intercourse
    - Use of alcohol and drugs
- What do you do to remember to start an on-demand PrEP regimen?
  - *Explore:*
    - Electronic resources (apps, alarms, online calendar, etc.)
    - Analog/behavioral resources (pills in sight, associating with meals, etc.)
  - *Explore:* whether these resources are used only for PrEP or for other continuously used medications
  - *Explore:* the positive and negative aspects of each mentioned strategy
- What do you do to remember to take the final pills of an on-demand PrEP regimen (+1+1)?
  - *Explore:*
    - Electronic resources (apps, alarms, online calendar, etc.)
    - Analog/behavioral resources (pills in sight, associating with meals, etc.)
  - *Explore:* whether these resources are used only for PrEP or for other continuously used medications
  - *Explore:* the positive and negative aspects of each mentioned strategy

- What do you do when you forget or delay a dose of on-demand PrEP?
    - *Explore:* behavioral changes and pill usage
- How do you usually transition between taking on-demand PrEP and daily PrEP?
    - *Explore:*
        - Frequency of sexual relations
        - Risk perceptions

Part Three: New technologies to support on-demand PrEP use (20 – 25 min.)

- Imagine you were invited to give suggestions and ideas to a team developing an application to help PrEP users take their pills.
    - What ideas or suggestions would you give?
        - *Explore:*
            - Security
            - Privacy (camouflage, access password, etc.)
            - Internet consumption
            - Availability on different OS (Android, iOS, Windows, etc.)
            - Design (colors, style, etc.)
            - Online and offline?
            - User identification (does it need login?)
            - Standalone or embedded in another app (chatbot, for example)

Part Four: What would an ideal PrEP service look like? (20 – 25 min.)

- For you, what would an ideal PrEP service look like?
    - *Encourage:*
        - Reflections on how pill pick-up would occur
        - Frequency of sample collections for tests
        - Frequency of consultations
        - Location of consultations
        - Support for diagnosis and treatment of STIs
        - Mental health support?

Finalizing

- Review the script. Would anyone like to add anything else?
