# Supplementary material for: Perceptions of Daily and On-Demand HIV Pre-Exposure Prophylaxis and Digital Adherence-Support Needs Among Cisgender Men in Brazil: Qualitative Interview and Focus Group Study
Source: J Med Internet Res. 2025 Oct 2;27:e66848. doi: 10.2196/66848 (PMC12492313; doi:10.2196/66848)
Supplement: Multimedia Appendix 2 [file jmir-v27-e66848-s002.pdf]

Supplemental Table 1. PrEP use trajectories, regimen preference, and adherence experiences of interview participants (n=19), five Brazilian cities, 2022–2023.

Codename	PrEP Use and Switching Trajectory	Regimen Preference and Motivations	Key Adherence Challenges and Experiences
**Allan**	Started and remained on on-demand PrEP.	Prefers on-demand due to fear of side effects and long-term over-medication.	Needed PEP "several times" due to unplanned sex without prophylaxis.
**Carlos**	Started and remained on on-demand PrEP.	Prefers on-demand to avoid daily medication and due to fear of continuous use side effects.	Reports having missed one of the final doses due to a significant delay.
**Enzo**	Started and remained on on-demand PrEP.	Prefers on-demand because he did not want to take a pill "every day".	Has delayed a dose by 3 to 4 hours.
**Ernesto**	Switched from daily to on-demand PrEP.	Prefers on-demand as it is "less aggressive" (given his diabetes) and fits his predictable sexual life.	Describes himself as very methodical ("caxias"), stating he has never delayed or missed a dose.
**Guto**	Switched between daily and on-demand regimens multiple times.	Prefers the regimen that best fits his life context at the moment (flexibility).	States he is very methodical and does not recall significant delays or missed doses.
**Gilvan**	Switched from daily to on-demand PrEP; currently makes "micro-switches" to daily use when anticipating more sexual activity.	Prefers the flexibility of on-demand but opts for daily use to avoid restarting the 2+1+1 protocol repeatedly.	Missed a dose once when his routine was interrupted but had no sexual intercourse.
**Jileandro**	Started and remained on on-demand PrEP.	Prefers on-demand as it suits his planned or weekend encounters.	Reports having delayed a dose by 3 to 4 hours once.

**Jamerson**	Switched from on-demand to daily PrEP.	Preference changed with lifestyle; daily became more practical with increased sexual frequency.	Reports being very methodical and never having missed a PrEP dose.
**João**	Started and remained on on-demand PrEP.	Prefers on-demand as it fits his sexual activity pattern, which is concentrated on weekends.	Has delayed taking the medication by about an hour.
**Jorge**	Switched from on-demand to daily PrEP after starting a relationship with a partner living with HIV.	Preference changed with his relationship context; daily became the best option.	States he never missed a dose of the on-demand regimen.
**James**	Switched from on-demand to daily PrEP due to difficulty managing the schedule with unpredictable encounters.	Prefers daily PrEP, as his current lifestyle makes the on-demand regimen "too complicated to manage".	Found the on-demand regimen "harder to manage" than the daily one.
**Lauro**	Switched between regimens; moved from on-demand to daily to build a habit, then returned to on-demand.	Shows a preference for the daily routine, as he finds adherence easier.	Reports "great difficulty with adherence" to on-demand, with delays and completely forgetting the last dose "many times".
**Liandro**	Switched from daily to on-demand PrEP, where he remains.	Prefers on-demand because he believes it is "less aggressive to the body".	The main challenge is remembering to take the pills with him when leaving home.
**Lisandro**	Started with on-demand PrEP but switches to continuous use during periods of higher sexual activity.	Prefers the flexibility of on-demand, which allows for continuous use when needed.	Reports a single case of a 5-hour delay in taking the last dose in two years of use.
**Marcelo**	Started and remained on on-demand PrEP (with a brief switch to daily for clinical reasons).	Prefers on-demand, as he returned to this regimen after the period of daily use.	States he has a good memory and does not need an alarm, viewing the schedule as a "commitment".
**Mauro**	Started and remained on on-demand PrEP.	Prefers on-demand as it suits his needs and avoids daily medication.	Is very organized; reports having delayed a dose by one hour at most.

**Viana**	Started with on-demand PrEP; no longer on PrEP due to a monogamous relationship.	Prefers on-demand as it fits his risk profile (weekend exposure).	The main challenge was remembering the initial doses, as encounters were often unplanned.
**Wellington**	Switched from on-demand to daily PrEP.	Prefers daily PrEP, as the on-demand regimen was incompatible with his "frantic" and "improvised" sex life.	The difficulty was not forgetting but the logistics of taking the initial dose with the required advance notice.
